# Exploring the link between internet addiction and sleep quality and the implications of this relationship: a systematic review

**DOI:** 10.47626/2237-6089-2025-1086

**Published:** 2025-11-05

**Authors:** Simone Gazale, Natia Horato, Antonio E. Nardi, Anna Lucia King

**Affiliations:** 1 Universidade Federal do Rio de Janeiro Instituto DELETE – Detox Digital e Uso Consciente de Tecnologias, Instituto de Psiquiatria Rio de Janeiro RJ Brazil Instituto DELETE – Detox Digital e Uso Consciente de Tecnologias, Instituto de Psiquiatria (IPUB), Universidade Federal do Rio de Janeiro (UFRJ), Rio de Janeiro, RJ, Brazil.; 2 UFRJ Laboratório de Pânico e Respiração Rio de Janeiro RJ Brazil Laboratório de Pânico e Respiração (LABPR), IPUB, UFRJ, Rio de Janeiro, RJ, Brazil.

**Keywords:** Excessive internet use, dependence on screens, sleep quality

## Abstract

**Objective:**

The internet has become an important element in people's lives. The increasing use of smartphones and other electronic devices has promoted an increase in digital interactions, resulting in significant problems in the field of mental health. However, one of the implications of excessive internet use is impaired sleep quality, especially among users who use the internet before bed.

**Methods:**

We searched the PubMed, Web of Science and PsycINFO databases to identify articles that addressed the association between excessive internet use and sleep quality. Studies in which participants had a previous diagnosis of insomnia or used psychoactive substances were excluded.

**Results:**

The initial search resulted in a total of 3269 articles, 25 of which met the inclusion criteria. The results suggested an association between excessive internet use and sleep quality.

**Conclusion:**

Excessive internet use significantly compromises sleep quality, directly affecting users’ mental and physical health. This study highlights the importance of strategies that promote digital education to raise awareness about the risks of excessive use of screens.

**Systematic review registration:** PROSPERO, CRD 42024610542.

## Introduction

The development of the internet has revolutionized global communications and increased access to information. The benefits provided by technology, including unlimited access to the internet through smartphones and other electronic devices, have made social, cultural, and economic interactions more agile and efficient.^1^ According to The Global Digital Report 2024,^2^ the world's population reached 8.08 billion inhabitants, an increase of 0.9% compared with that in 2023. By the beginning of this year, the number of mobile phone users had reached 5.61 billion, which corresponds to 69.4% of the global population. Additionally, internet use increased by 1.8% compared with the beginning of 2023, with more than 66% of the world's population using the internet. The average daily time of internet use is approximately 6 hours and 36 minutes per person.^2^ In Brazil, the situation is even more serious, with the country ranking second in terms of screen time, registering an average of 9 hours and 13 minutes per day.^2^

Given the current global context, excessive use of the internet can lead to a series of impairments, including inadequate time management during the day and impairments in sleep quality at night. According to the World Health Organization,^3^ the use of the internet, computers, smartphones and other electronic devices has increased significantly in recent decades. This growth, while providing clear and considerable benefits to users, is also associated with cases of overuse, often resulting in negative health consequences.

Digital technology has become an indispensable part of everyday life, but excessive use can trigger digital addiction, which is characterized by the inability to reduce screen time despite negative effects.^4^ Furthermore, the absence of a cell phone, internet disconnection, or distancing from the computer can reach such a high degree that they compromise daily activities, generating typical symptoms of nomophobia, such as anxiety, discomfort, and panic, among others.^5^ Nomophobia is defined as feelings of distress, discomfort, or anxiety arising from the unavailability of any means of virtual communication, including not only cell phones but also personal computers, tablets, and other devices.^5^ One of the most impactful consequences of excessive screen use is the loss of sleep quality, which directly affects an individual's mental health.^6^

Research on the importance of sleep quality has emerged as a topic of great relevance, as it has become a significant burden for health systems.^6^ According to the National Sleep Foundation, the quality of sleep is crucial for obtaining physical, mental and emotional benefits during rest. Moreover, sleep quality is related to other aspects of sleep, such as sleep duration, satisfaction, and regularity.^7^ Sleep quality is measured through tests and technologies and is generally divided into four main dimensions: sleep latency, which refers to the time it takes to fall asleep; awakening, which is the number of times sleep interruptions occur during the night; waking after the onset of sleep, which refers to the interval of time in which the person remains awake after falling asleep; and sleep efficiency, which is the ratio of effective sleep time to total time lying in bed.^6^

Recent studies have indicated that individuals who use smartphones before bed have greater evidence of a decrease in sleep quality.^8^ Exposure to screens before bed has been shown to impair not only sleep quality but also several other factors related to sleep deprivation, such as chronic tiredness, daytime sleepiness, lack of appetite, memory deficit, and decreased levels of attention and concentration. Studies suggest that light is one of the main factors responsible for the synchronization of the circadian cycle, although nonphotonic signals, such as mealtimes, physical activity, and social interactions, also play important roles in regulating this cycle.^9^

In view of these findings, strategies such as sleep hygiene practices, digital education, and parental guidance in children and adolescents have been recommended to mitigate the effects of excessive use of electronic devices on sleep quality. Therefore, this systematic review aims to critically analyze the available literature on the association between excessive internet use and sleep quality, with a focus on identifying the clinical implications of this relationship. By gathering current evidence, we seek to contribute to the understanding of the risks associated with the problematic use of digital technologies, elucidate the implications of this behavior and its impacts on sleep.

## Methodology

This systematic review was reported according to the Preferred Reporting Items for Systematic Reviews and Meta-Analyses guidelines (PRISMA)^10^ guidelines and was registered in the PROSPERO database under the registration number CRD 42024610542.

A comprehensive search strategy was used on the basis of the PICOS^11^ framework. The systematic review included individuals with impairments in sleep quality who reported excessive use of digital technology. The comparators included individuals who were conscientious about their use of technology. The outcome was the impact of technology use on sleep quality. There were no restrictions regarding study design.

The PubMed, Web of Science, and PsycINFO databases were systematically searched using the following keywords: Internet addiction OR Smartphone addiction OR Digital dependency OR Technology overuse OR Online addiction OR Excessive screen time AND Sleep quality OR Insomnia OR Sleep disturbance OR Poor sleep OR Sleep problems OR Sleep issues OR Sleep disorders. The inclusion criteria for this study included adults of both genders, users of technologies and individuals who underwent sleep quality assessment. Studies whose participants had a previous diagnosis of insomnia or who used psychoactive substances, such as energy drinks containing caffeine, were excluded. Two reviewers independently searched all databases, and there were no restrictions on publication date or language. After removing duplicate records, S.G. and N.H. identified the relevant articles based on their titles and abstracts, followed by screening the full texts of the selected studies according to the inclusion criteria. Discrepancies in judgment were thoroughly discussed, and consensus was reached or resolved by a third reviewer (A.L.K.).

Although this review followed the PRISMA guidelines and identified a considerable number of studies with relevant results, a meta-analysis was not possible due to the high methodological heterogeneity among the included studies. The instruments used to assess both excessive internet use and sleep quality varied significantly between studies, making it impossible to quantitatively group the data reliably.

### Methodological evaluation and data extraction

The methodological quality of the studies included in the review was assessed via the Joanna Briggs Institute (JBI) Checklist.^12^ The JBI offers validated checklists for the quality assessment of different types of observational studies, including cross-sectional, cohort, and case-control studies. The assessment of risk of bias was performed by two independent reviewers, who applied the appropriate checklists according to the type of study included in the review and covered aspects such as clarity of inclusion criteria, validity of exposure and outcome measures, control of confounders, and adequate statistical analysis. The evaluation was carried out by two independent reviewers, who applied the appropriate checklists according to the type of study included in the review. The following data were extracted: first author, sample size, type of technology used, sleep quality assessment instrument and excessive internet use, as well as the individual outcome of each article. Data extraction and methodological evaluation were conducted by two reviewers (S.G. and N.H.) and verified by a third reviewer (A.L.K.) (Supplementary Table S1).

## Results

**Figure 1:**
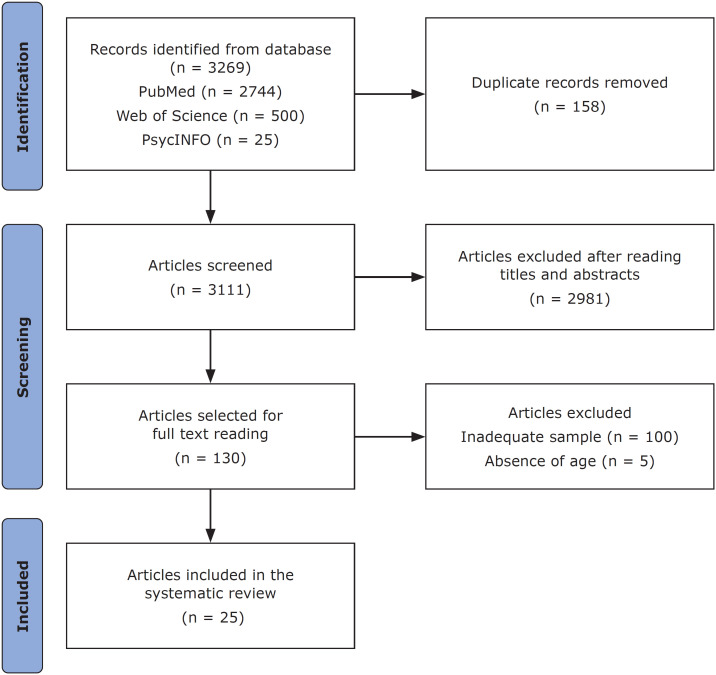
PRISMA flow diagram of the search and selection process.

A total of 3269 articles were initially retrieved from the databases. A total of 158 duplicates were identified and removed. After the titles and abstracts were screened, 2981 articles were removed, and 130 studies were included for full-text screening. Subsequently, 105 studies were excluded because they did not meet the eligibility criteria, and thus, 25 articles were ultimately included in this systematic review (Figure 1). The synthesis of the data extracted from the included articles is presented in Table 1.

**Table 1:** Summary of the articles included in the systematic review

Study	Total sample	Male	Female	Acts (Sd)	Assessment instruments	Technology devices
Acikgoz et al.,^13^ 2022	910	461	449	15.7 (1.2)	s-IAT; SAS; PSQI	Smartphone
Karki et al.,^14^ 2021	390	216	174	15.0 (1.0)	PSQI; IAT	Smartphone/Laptop
Çelebioglu et al.,^15^ 2020	1487	901	586	16.6 (1.9)	PSQI; YIAT-SF	Desktop computers/mobile devices
Chi et al.,^16^ 2022	1647	784	863	13.8 (0.9)	KIDSSCREEN-27; PSQI-K; S-SCALE	Smartphone
Caumo et al.,^17^ 2019	177	62	115	15.5 (1.7)	PSQI; MCQ	Computer, tablets, video games, Smartphone
Wang et al.,^18^ 2021	1040	416	624	20.3 (1.4)	PSQI; IAT; GAS	Video game
Park et al.,^19^ 2022	4287	1942	1837	10.5 (2.5)	SAPS	Smartphone
Guclu et al.,^20^ 2023	1959	925	1034	17.5 (2.5)	PSQI; YIAT-SF; SAS-SV	Smartphone
Hidayatullah et al.,^21^ 2023	600	365	235	16.2 (1.4)	PSQI; IAT	Smartphone
Nikolic et al.,^22^ 2023	761	240	521	21.8 (2.1)	PSQI; SAS-SV; DASS-21	Smartphone
Zhuang et al.,^23^ 2023	2661	1153	1508	19.9 (1.2)	PSQI; It does; BSMAS	Smartphone
Qanash et al.,^24^ 2021	608	171	437	29.5 (10.5)	PSQI; SAS-SV	Electronic Device
Sanusi et al.,^25^ 2022	420	103	317	20.9 (1.6)	PSQI; SAS; PSS-10	Smartphone
Alzhrani et al.,^26^ 2023	773	312	461	25.9 (8.3)	PSQI; SABAS; SQS	Smartphone
Hasan et al.,^27^ 2023	552	113	439	21.2 (5.1)	PSQI; SAS-SV; MEQ	Smartphone
Saman et al.,^28^ 2018	321	281	40	21.3 (1.8)	PSQI; COS	Cell phone/Smartphone
Li et al.,^29^ 2021	2298	1108	1180	13.3 (2.3)	PSQI; SSE	Smartphone
You et al.,^30^ 2020	1104	408	696	20.2 (1.4)	PSQI; YIAS-8	Smartphone
Correa-Iriarte et al.,^31^ 2023	313	165	148	30.5 (10.1)	PSQI; BPS SAS-SV	Smartphone
Gupta et al.,^32^ 2020	222	152	70	20.7 (2.2)	PSQI; YIAT	Smartphone
Lane et al.,^33^ 2021	422	79	343	20.2 (2.3)	CPSQI; SAPS	Smartphone
Zhu et al.,^34^ 2023	2744	845	1899	20.8 (2.3)	PSQI; SMF; SMA FOMO	Smartphone
Alahdal et al.,^35^ 2023	373	246	127	15.8 (5.5)	PSQI; SAS-SV	Smartphone
Andhi et al.,^36^ 2022	310	77	230	29.5 (10.5)	PSQI	Smartphone
Wu et al.,^37^ 2021	4325	1668	2657	19.9 (1.3)	CPSQI; SAS-SV	Smartphone

BSMAS = The Bergen Social Media Addiction Scale; COS = Cell Phone Overuse Scale; CPSQI = Chinese Pittsburgh Sleep Questionnaire Index; DASS-21 = Depression, Anxiety, and Stress Scale-21 items; FAS = Fatigue Assessment Scale; FOMO = Fear of Missing Out; GAS = Gaming Addiction Scale; IAT = Internet Addiction Test; KIDSCREEN 27 = Health-Related Quality of Life Questionnaire; MCQ = Munich Chronotype Questionnaire; PSQI = Pittsburgh Sleep Quality Index; PSQI-K = Korean version of the Pittsburgh Sleep Quality; PSS-10 = Perceived Stress Scale-10; SABAS = Six-Item Smartphone Application-Based Addiction Scale; SAPS = Smartphone Addiction Proneness Scale; SAS = Smartphone Addiction Scale; SAS-SV = Smart-Phone Addiction Survey–Short Version; s-IAT = Short Internet Addiction Test; SMA = Social Media Addiction; SMF = Social Media Fatigue; SQS = Sleep Quality Scale; S-SCALE = Smartphone Addiction Self-Diagnosis Scale; SSE = Smartphone Self-Efficacy; YIAT = Young's Internet Addiction Test; YIAS-8 = Young's 8-item Internet Addiction Diagnosis Questionnaire; YIAT-SF = Young's Internet Addiction Test-Short Form.

All studies included in this systematic review presented individual findings that suggest an association between excessive internet use and sleep quality in different contexts. Among adolescents, excessive internet use associated with low sleep quality was due to excessive use of smartphones.^13^ Age was also found to be a relevant factor in this association. Older individuals showed worsening sleep quality associated with internet use.^14^ However, even among adolescents who make moderate use of electronic devices but use the devices before bedtime, sleep quality was also impaired.^15^ Additionally, a study conducted during the COVID-19 pandemic suggested that increased screen time aggravated inappropriate smartphone use.^16^ Therefore, cell phones have emerged as the most commonly used devices during the day, especially before bedtime.^17^ Wang et al.^18^ also highlighted that addiction to electronic games has a direct negative influence on feelings of anguish, which can lead to a decline in the quality of sleep among adolescents. Park et al.^19^ suggested that children who spend less time with their parents are at greater risk of excessive internet use.

According to studies conducted by Guclu et al.^20^ and Hidayatullah et al.,^21^ sleep is a fundamental daily activity for the quality of life, mental health and psychological well-being of individuals in the academic environment. This is because during sleep, the brain consolidates information learned during the day, thus transforming short-term memories into long-term memories. In this context, Nikolic et al.^22^ and Zhuang et al.^23^ reported that 21% of students who used smartphones for more than 4 hours experienced a decrease in sleep quality and increased levels of anxiety, fatigue, attention and impaired cognitive functions. An additional study conducted by Qanash et al.^24^ with academics in the health area reported that 98.84% of participants used smartphones before bed and that only 44.57% put them silent.

Sanusi et al.^25^ also reported that although the excessive use of electronic devices is common among students, 33.3% of the research participants used smartphones for more than 6 hours without academic connotations, which was directly linked to the worsening of sleep quality. Thus, dependence on smartphones appears was negatively correlated with worsening sleep quality^26,27^ and the use of social networks.^28^

Some studies have also shown sex differences in the association between excessive internet use and sleep quality. Chi et al.^16^ suggested that girls are at greater risk of developing mental health problems associated with impaired sleep quality and screen addiction. Li et al.^29^ reported that boys use smartphones longer during the day and that girls use them mainly before bed. Additionally, boys are considered to be more predisposed to exhibiting habitual use of technologies, which negatively impacts issues related to memory and sleep quality.^30^ Correa-Iriarte et al.^31^ noted that women are more likely to use smartphones for longer and for social purposes (social networks and messages). Gupta et al.^32^ investigated the prevalence of excessive internet use among men and women and reported that the rates of excessive internet use were 20.4% and 12.9%, respectively.

Similar behaviors related to the use of electronic devices and changes in sleep quality, due to sex differences, are presented in the study by Lane et al.^33^ The study also suggests that possible neurochemical mechanisms that connect personality traits to smartphone addiction contribute to the discovery of theoretical models on smartphone addictions, providing reflections on prevention and intervention aimed at reducing addiction to mobile devices.^33^

## Discussion

**Figure 2:**
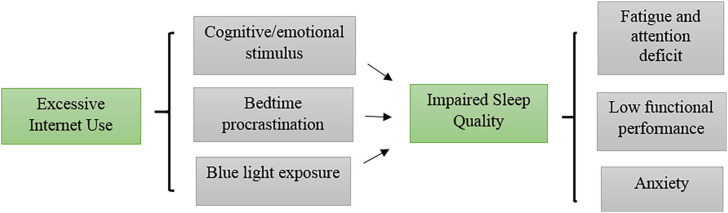
Conceptual diagram illustrating how excessive internet use is linked to poor sleep quality through cognitive and emotional stimulation, bedtime procrastination, and blue light exposure, resulting in fatigue, impaired performance, and anxiety.

The purpose of this study was to analyze the impact of excessive internet use on sleep quality, in order to understand the risks associated with the problematic use of digital technologies and the clinical implications of this behavior on the physical and mental health of users^37^ (Figure 2).

### Behavioral and physiological factors

The relationship between sleep quality and quantity is complex because it involves multiple interconnected factors, such as biology and lifestyle habits. However, both are essential components of physical and mental well-being. In fact, inadequate sleep causes daytime tiredness, mood swings, and possible states of anxiety, anguish, and depressive symptoms.^21^ Therefore, even sleeping the recommended number of hours, poor sleep architecture can harm an individual's health.^38^

According to Rafael Pelayo,^39^ to assess sleep quality, the quantity, quality, sleep schedule (which refers to the time when the person lies down to sleep) and the individual's mental state are considered. In this sense, technological dependence is associated with poorer sleep quality, especially among adolescents and young adults. This is because these groups use screens for longer periods at night and tend to have less structured sleep routines, thus compromising physical and cognitive development, which is vulnerable in these age groups.^39^

In addition to behavioral factors, physical aspects also play important roles in the deterioration of sleep quality and represent a gap in this study. The light emitted by smartphones, for example, inhibits the production of melatonin, which impairs the induction and maintenance of sleep.^40^ During the day, light waves contribute to increased attention. However, at night, its effects are associated with impaired sleep quality, as well as reduced cognitive performance, thereby reducing nocturnal melatonin synthesis in the pineal gland and causing a circadian cycle misalignment.^40^ However, this physiological mechanism can be circumvented through strategies that reduce light emission or the use of sleep-inducing medications, which was not considered in this study.^40^

The interactive and often stimulating nature of online activities also contributes to the difficulty in mentally disconnecting before bed. Compulsive use of apps and social networks leads to prolonged screen time and can generate cognitive and emotional arousal effects. Prolonged exposure to digital notifications and stimuli reduces sleep time and increases the risk of bedtime procrastination.^41^ In studies in which the average time spent on smartphones is approximately six hours per day, students realized that they use the devices for longer than they intended to, due to the constant checking of app notifications.^34,35^ Thus, Wu et al.^36^ suggested that reducing the use of electronic devices with internet access favors the reduction of anxiety symptoms.^34^

### Age and gender differences

The studies included in this review indicated important variations in patterns of technology use according to age and gender. Adolescents and university students were the most represented groups in the samples, with heavy use of smartphones at night. Adolescents who use electronic devices before bedtime demonstrate worse sleep quality even with moderate use.^34,41^

Regarding gender, variations in the patterns of technology use were observed. Females tend to use devices before bedtime, often in activities related to social networks, while males demonstrate more generalized patterns of use, without discrimination of time or type of content, and are more predisposed to develop habitual use of technologies.^32^ Despite these differences, prolonged use of devices before bed was harmful for both groups.^40^

### Limitations

Despite the consistency of the findings, this review has some limitations. Most studies have a cross-sectional design, which limits causal inference. In addition, there is a lack of studies with economically active adult populations, such as workers who use digital technologies in the professional context, whose impacts on sleep can be aggravated by the mental load associated with work tasks.^42^ There is also a lack of longitudinal studies and experimental interventions that can test strategies to reduce digital use and their repercussions on sleep quality. There is still a lack of specific research on the type of content accessed (entertainment, social networks, work) and its differential impact on sleep.^42^ Considering the post-pandemic context, in which screen time has increased substantially,^16^ further research is needed to understand behavioral changes and their lasting effects.

An important limiting factor was the impossibility of performing a meta-analysis. The included studies used different scales to assess problematic use of the internet and different sleep assessment instruments, with different cultural versions and application methodologies. This heterogeneity undermines direct comparability between results and limits the possibility of calculating combined effect estimates. For future studies, greater standardization of the instruments used is recommended, which could allow for more robust quantitative analyses.

## Conclusion

The evidence presented in this study suggests that excessive use of electronic devices to access the internet, especially before bedtime, is considered a risk factor for impaired sleep quality, which can negatively affect users’ physical and mental health. These findings reinforce the importance of interventions aimed at promoting sleep health among users of digital technologies. Sleep hygiene strategies, such as avoiding exposure to light from screens before bed, maintaining regular bedtimes and getting up, and limiting the use of devices in the sleep environment, can be effective.

In addition, digital education actions and specific guidelines for parents, educators, and health professionals should be encouraged, especially in young populations. Screening for problematic use of technologies may become a relevant clinical practice, considering its association with sleep disturbances and mental health symptoms.

## Trends in Psychiatry and Psychotherapy - Supplementary Material



## Data Availability

The data that support this study are available from the authors upon request.
