# Supplementary material for: Exploring the link between internet addiction and sleep quality and the implications of this relationship: a systematic review
Source: Trends Psychiatry Psychother. 2025 Nov 5;47:e20251086. doi: 10.47626/2237-6089-2025-1086 (PMC12956153; doi:10.47626/2237-6089-2025-1086)
Supplement: Supplementary file 1 [file 2238-0019-trends-47-e20251086-suppl01.pdf]

http://doi.org/10.47626/2237-6089-2025-1086

Table S1 - Joanna Briggs Institute (JBI): critical-analytical cross-cross studies

Study	Criteria for inclusion in the sample clearly defined	Subjects and the setting described in detail	Exposure measured in a valid and reliable way	Objective, standard criteria used for measurement of the condition	Confounding factors identified	Strategies to deal with confoundin g factors stated	Outcomes measured in a valid and reliable way	Appropriate statistical analysis used	Total score
Saman et al., 2018	*	*	*	*	*	*	*	*	8
Caumo et al., 2019	*	*	*	*			*	*	6
Çelebioglu et al., 2020	*	*	*	*	*	*	*	*	8
Gupta et al., 2020	*	*	*	*	*		*	*	7
You et al., 2020	*	*	*	*	*		*	*	7
Wu et al., 2021	*	*	*	*	*	*	*	*	8
Qanash et al., 2021	*	*	*	*	*	*	*	*	8
Li et al., 2021	*	*	*	*			*	*	6
Karki et al., 2021	*	*	*	*	*		*	*	7
Lane et al., 2021	*	*	*	*	*	*	*	*	8
Wang et al., 2021	*	*	*	*			*	*	6
Andhi et al., 2022	*	*	*	*	*		*	*	7
Acikgoz et al., 2022	*	*	*	*	*	*	*	*	8
Chi et al., 2022	*	*	*	*	*		*	*	7
Park et al., 2022	*	*	*	*	*	*	*	*	8
Sanusi et al., 2022	*	*	*	*	*	*	*	*	8
Nikolic et al., 2023	*	*	*	*	*	*	*	*	8
Zhuang et al., 2023	*	*	*	*	*		*	*	7
Zhu et al., 2023	*	*	*				*	*	5
Alahdal et al., 2023	*	*	*	*			*	*	6
Alzhrani et al., 2023	*	*	*	*	*	*	*	*	8
Hasan et al., 2023	*	*	*	*	*	*	*	*	8
Correa-Iriarte et al., 2023	*	*	*	*	*		*	*	7
Hidayatullah et al., 2023	*	*	*	*	*	*	*	*	8
Guclu et al., 2023	*	*	*	*	*	*	*	*	8
